# Comparison of ^18^F-fluorothymidine Positron Emission Tomography/Computed Tomography and ^18^F-fluorodeoxyglucose Positron Emission Tomography/Computed Tomography in Patients with Breast Cancer

**DOI:** 10.3390/tomography8050211

**Published:** 2022-10-11

**Authors:** Mio Mori, Tomoyuki Fujioka, Ryota Ichikawa, Reina Inomata, Leona Katsuta, Yuka Yashima, Emi Yamaga, Junichi Tsuchiya, Kumiko Hayashi, Yuichi Kumaki, Goshi Oda, Tsuyoshi Nakagawa, Iichiroh Onishi, Kazunori Kubota, Ukihide Tateishi

**Affiliations:** 1 Department of Diagnostic Radiology, Tokyo Medical and Dental University, 1-5-45 Yushima, Bunkyo-ku, Tokyo 113-8510, Japan; 2 Department of Surgery, Breast Surgery, Tokyo Medical and Dental University, 1-5-45 Yushima, Bunkyo-ku, Tokyo 113-8510, Japan; 3 Department of Comprehensive Pathology, Tokyo Medical and Dental University, 1-5-45 Yushima, Bunkyo-ku, Tokyo 113-8510, Japan; 4 Department of Radiology, Dokkyo Medical University Saitama Medical Center, 2-1-50 Minamiko-shigaya, Koshigaya, Saitama 343-8555, Japan

**Keywords:** breast cancer, ^18^F-fluorothymidine, ^18^F-fluorodeoxyglucose, positron emission tomography

## Abstract

The uptake of ^18^F-fluorothymidine (^18^F-FLT) depends on cells’ proliferative rates. We compared the characteristics of ^18^F-FLT positron emission tomography/computed tomography (PET/CT) with those of ^18^F-fluorodeoxyglucose (^18^F-FDG) PET/CT for breast cancer. We prospectively diagnosed patients with breast cancer who underwent ^18^F-FLT PET/CT and ^18^F-FDG PET/CT. Subsequently, significant differences and correlation coefficients of the maximum standardized uptake value (SUVmax) in primary breast cancer and axillary lymph nodes were statistically evaluated. We enrolled eight patients with breast cancer. In six treatment-naive patients, the SUVmax for primary lesions showed a significant difference (mean, 2.1 vs. 4.1, *p* = 0.031) and a strong correlation (*r* = 0.969) between ^18^F-FLT and ^18^F-FDG. Further, although the SUVmax for the axillary lymph nodes did not show a significant difference between ^18^F-FLT and ^18^F-FDG (P = 0.246), there was a strong correlation between the two (r = 0.999). In a patient-by-patient study, there were cases in which only ^18^F-FDG uptake was observed in lymph nodes and normal breasts. Bone metastases demonstrated lower accumulation than bone marrow on the ^18^F-FLT PET/CT. In conclusion, a strong correlation was observed between the ^18^F-FLT PET/CT and ^18^F-FDG PET/CT uptake. Differences in the biochemical characteristics of ^18^F-FLT and ^18^F-FDG were reflected in the accumulation differences for breast cancer, metastatic lesions, and normal organs.

## 1. Introduction

Breast cancer staging aids in determining disease severity, informing treatment planning, and predicting prognosis [1]. ^18^F-fluorodeoxyglucose positron emission tomography/computed tomography (^18^F-FDG PET/CT) can be used to evaluate a lesion’s morphology and glucose metabolism [2]. In addition, the maximum standardized uptake value (SUVmax) of ^18^F-FDG can inform patients’ prognosis [3,4,5]. However, ^18^F-FDG accumulates in areas of inflammation and benign/malignant neoplasms [6]; considerable experience and knowledge are necessary when using ^18^F-FDG to achieve an accurate diagnosis.

Recently, 3′-deoxy-3′-^18^F-fluorothymidine (^18^F-FLT) was introduced as a stable cell proliferation imaging agent [7]. This pyrimidine analog uses the DNA synthesis salvage pathway for imaging proliferation [8]. The uptake of ^18^F-FLT reflects the activity of thymidine kinase-1, an enzyme expressed during the DNA synthesis phase of the cell cycle [9]. ^18^F-FLT PET is often compared with ^18^F-FDG PET for the visualization, diagnosis, and staging of lung, head, neck, stomach, esophagus, brain, and breast tumors [9,10,11,12,13,14,15,16]. Although the tumoral uptake of ^18^F-FLT is generally lower than that of ^18^F-FDG, ^18^F-FLT PET can provide high specificity because it is less likely to accumulate in the areas of inflammation associated with cancer therapy [6,8,17]. Furthermore, ^18^F-FLT uptake correlates significantly with cell proliferation, as indicated by the Ki-67 labeling indices in lung and brain tumors [6,9,13]. This tracer can help differentiate benign from malignant lesions, measure tumor aggressiveness, and monitor treatment response [6,8,18]. Few studies have compared ^18^F-FLT PET/CT and ^18^F-FDG PET/CT in patients with untreated breast cancer [19]. However, several studies have used ^18^F-FLT PET/CT to assess the early response to hormone therapy and chemotherapy as well as long-term survival in patients with breast cancer treated with neoadjuvant chemotherapy [18,20,21,22]. In this study, we aimed to clarify the characteristics of these tracers by comparing ^18^F-FLT PET/CT and ^18^F-FDG PET/CT in patients with breast cancer and assess the role of ^18^F-FLT PET/CT in breast cancer diagnosis.

## 2. Materials and Methods

### 2.1. PET/CT Protocol

^18^F-FLT and ^18^F-FDG were administered intravenously to patients at a dose of 3.7 MBq/kg. Fasting for at least 4 h was required before ^18^F-FDG administration but not for ^18^F-FLT administration. Whole-body images were obtained using a PET/CT system (Cartesion Prime or Celesteion; Canon Medical Systems, Tochigi, Japan). No-contrast CT scans were performed using the following parameters: pitch, 0.938; gantry rotation time, 0.5 s; table time, 30 mm/s; auto-exposure control (SD 20), 120 KVp; and slice thickness, 2.0 mm. After approximately 60 min of ^18^F-FDG administration, a whole-body emission PET was performed using the following parameters for the Cartesion Prime and Celesteion, respectively: emission time per bed, 90 s and 2 min; bed position, 6–7 and 9–10; slice thickness, 2.11 and 4.08 mm; and matrix, 336 × 336 and 144 × 144.

### 2.2. PET/CT Analysis

One of four nuclear medicine specialists with 8–12 years of experience prospectively evaluated eligible patients with breast cancer. The nuclear medicine specialists, who were unblinded to each patient’s breast cancer diagnosis, evaluated the images and cross-referenced the mammography, ultrasound, and magnetic resonance imaging images as required. Accumulations within primary breast tumors and metastatic lesions were measured in terms of the SUVmax using Vox-base version 2.8 (J-MAC System, Inc., Hokkaido, Japan). For diagnosing the axillary lymph node metastases, we compared the tumor size and ^18^F-FDG uptake between the affected and contralateral sides. A mass with an abnormal ^18^F-FDG uptake was considered a distant metastasis. Concurrent CT scans were also carefully examined for regions with high physiologic accumulation (i.e., the brain for ^18^F-FDG PET/CT and bone marrow for ^18^F-FLT PET/CT).

### 2.3. Statistical Analysis

The SUVmax for primary breast cancer and axillary lymph nodes in treatment-naive (no prior surgery or chemotherapy) patients before PET/CT were compared between ^18^F-FLT PET/CT and ^18^F-FDG PET/CT. Distributions were analyzed using the Shapiro–Wilk test, and *p*-values of ≥0.05 were considered normally distributed. Significant differences were tested using a two-tailed paired *t*-test for normally distributed variables and the Wilcoxon signed-rank test for non-normally distributed variables. A *p*-value of <0.05 was considered statistically significant. In addition, the correlation between the SUVmax of the ^18^F-FLT PET/CT and that of the ^18^F-FDG PET/CT was assessed using Pearson’s correlation coefficient. The correlation coefficients ranged from −1 to +1, with 0 indicating no linear or monotonic associations; the relationship was considered stronger as the coefficient approached an absolute value of 1 [23].

## 3. Results

A total of 8 female patients with breast cancer, with a mean age of 64.8 years (standard deviation, 9.8 years; range, 49–73 years), were enrolled. Six patients were treatment-naive before the PET/CT, which included three surgical cases and three inoperable cases. Table 1 shows the number of days from biopsy to ^18^F-FLT PET/CT, ^18^F-FDG PET/CT, and surgery. All six treatment-naive breast cancers were invasive ductal carcinomas (the luminal type). We compared the ^18^F-FLT PET/CT with the ^18^F-FDG PET/CT in one of the two patients treated (i.e., non-treatment-naive) before the PET/CT. The interval between the PET/CTs was four days. Further, another patient was assessed solely based on recent ^18^F-FLT PET/CT findings.

### 3.1. Surgical Cases

**Figure 1:**
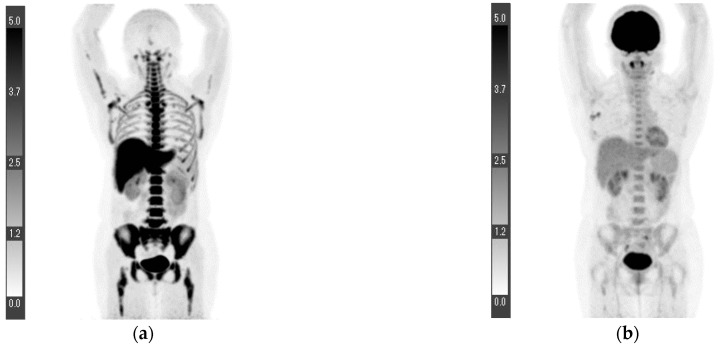

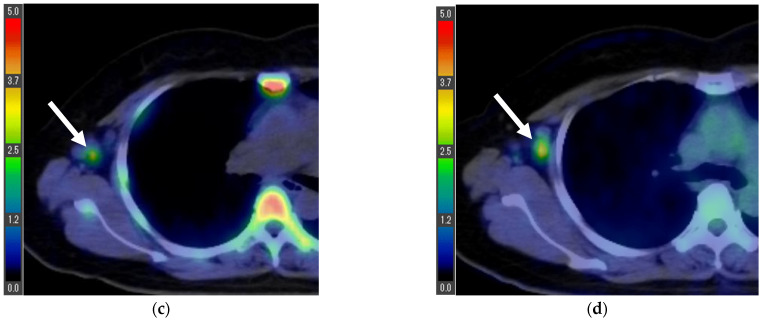
Maximum intensity projection (MIP) of ^18^F-FLT PET (**a**), MIP of ^18^F-FDG PET (**b**), axial image of ^18^F-FLT PET/CT (**c**), and axial image of ^18^F-FDG PET/CT (**d**). The axillary lymph node that showed the greatest accumulation differed between the ^18^F-FLT PET/CT and ^18^F-FDG PET/CT, with SUVmax values of 3.0 and 3.4, respectively (arrows). Patient 1 (Figure 1).

Three patients underwent ^18^F-FLT PET/CT for preoperative staging. In all patients, the SUVmax values of the primary lesions and axillary lymph nodes were lower on the ^18^F-FLT PET/CT than on the ^18^F-FDG PET/CT (Table 2). Patient one had metastases in 5/15 dissected axillary lymph nodes. The axillary lymph node that showed the greatest accumulation differed between the ^18^F-FLT PET/CT and ^18^F-FDG PET/CT, with SUVmax values of 3.0 and 3.4, respectively (Figure 1).

### 3.2. Inoperable Cases

**Figure 2:**
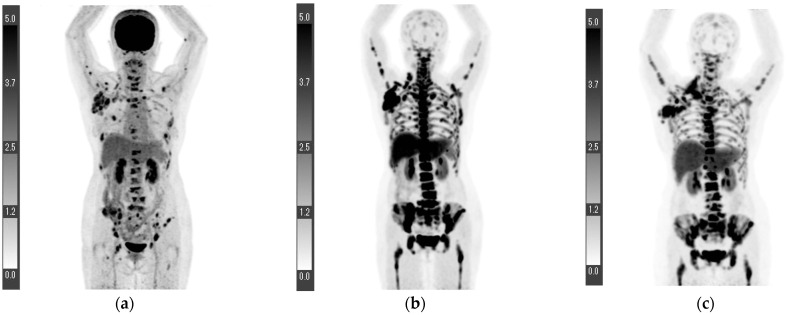

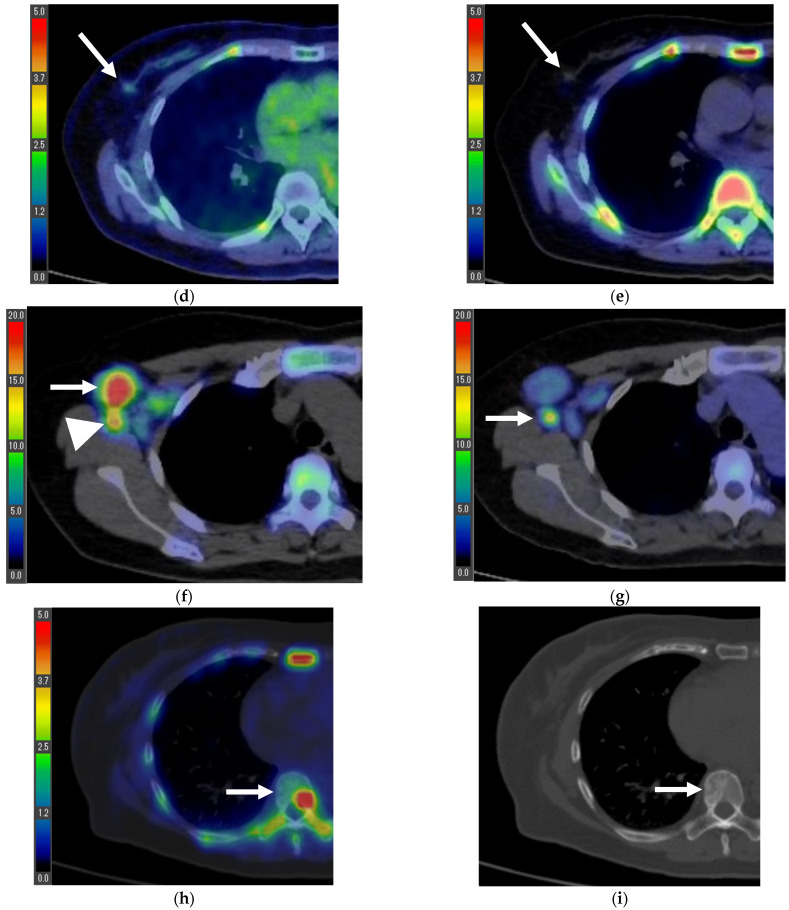
First maximum intensity projection (MIP) of ^18^F-FDG PET (**a**), MIP of ^18^F-FLT PET performed 3 days later (**b**), and MIP of ^18^F-FLT PET performed 5 months later (**c**). In the axial image of the PET/CT at the slice of the primary right breast cancer, both the ^18^F-FDG PET/CT and ^18^F-FLT PET/CT showed trace accumulation ((**d**,**e**), arrows). The axillary lymph node with the greatest accumulation differed between the ^18^F-FLT PET/CT (**f**) and ^18^F-FDG PET/CT (**g**), and the SUVmax values were 30.0 and 14.2, respectively (arrows). The lymph node corresponding to the node with the greatest accumulation on the ^18^F-FDG PET/CT showed an SUVmax of 16.7 on the ^18^F-FLT PET/CT ((**f**), arrowhead). The post-chemotherapy thoracic spine metastasis showed low ^18^F-FLT accumulation and bone sclerosis, suggesting that cell proliferation had decreased ((**h**,**i**), arrows). Patient 4 (Figure 2).

Three patients possessed an inoperable disease. The SUVmax of their primary lesions was lower on the ^18^F-FLT PET/CT than on the ^18^F-FDG PET/CT (Table 2). In patient four, both the initial ^18^F-FLT and ^18^F-FDG PET/CTs showed a slight accumulation in the primary lesion but high accumulation in multiple lymph node metastases, as well as in multiple bone metastases. She underwent chemotherapy, and a second ^18^F-FLT PET/CT was performed 147 days after the first ^18^F-FLT PET/CT. At that time, some bone metastases showed lower ^18^F-FLT accumulation than the physiological accumulation within the bone marrow, presumably because the treatment reduced cell proliferation (Figure 2).

**Figure 3:**
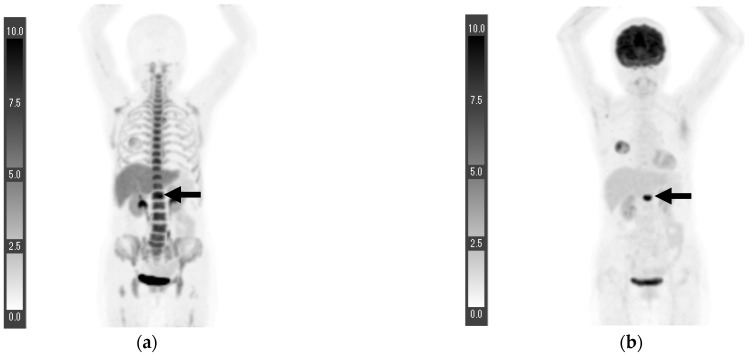
Maximum intensity projection (MIP) of ^18^F-FLT PET (**a**) and MIP of ^18^F-FDG PET 25 days before the ^18^F-FLT PET/CT (**b**). The patient had a first lumbar vertebra metastasis, and the SUVmax of the ^18^F-FLT PET/CT and ^18^F-FDG PET/CT were 10.8 and 12.6, respectively (arrows). Patient 6 (Figure 3).

Patient six experienced a first lumbar vertebra metastasis, and ^18^F-FDG PET/CT revealed a clear accumulation. The SUVmax of the first lumbar vertebra was 12.6, whereas that of the second lumbar vertebra was 1.9 (Figure 3). As for the ^18^F-FLT PET/CT, which was performed 25 days before the ^18^F-FDG PET/CT, the accumulation in the first lumbar vertebra (SUVmax, 10.8) was similar to the bone marrow uptake (SUVmax in the second lumbar vertebra, 8.5), but the CT showed an osteolytic mass (not shown).

### 3.3. Statistical Analysis

**Figure 4:**
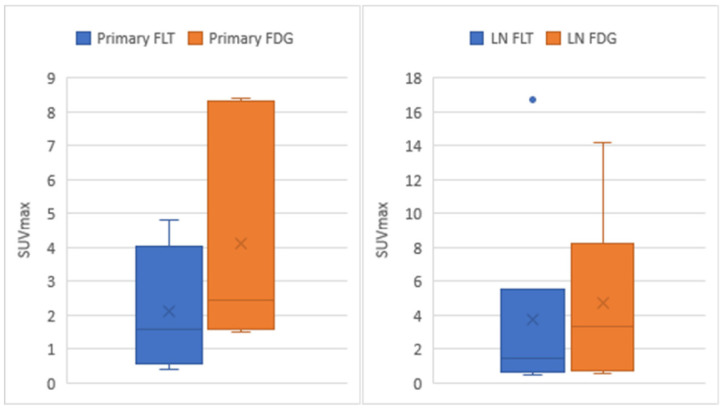
Boxplots of the SUVmax for treatment-naive patients (patients 1–6). SUVmax, maximum standardized uptake value; primary FLT, SUVmax of the primary breast cancer on ^18^F-fluorothymidine positron emission tomography/computed tomography (^18^F-FLT PET/CT); primary FDG, SUVmax of the primary breast cancer on ^18^F-fluorodeoxyglucose positron emission tomography/computed tomography (^18^F-FDG PET/CT); LN FLT, SUVmax of the axillary lymph node on ^18^F-FLT PET/CT; LN FDG, SUVmax of the axillary lymph node on ^18^F-FDG PET/CT.

When the Shapiro–Wilk test was performed for treatment-naive patients with primary breast cancer (patients 1–6), the *p*-value for the SUVmax was 0.596 for the ^18^F-FLT PET/CT and 0.061 for the ^18^F-FDG PET/CT, and both were normally distributed. Regarding the subsequent two-tailed paired *t*-test, the *p*-value was 0.031, and the SUVmax of the ^18^F-FLT PET/CT was significantly lower than that of the ^18^F-FDG PET/CT (mean, 2.1 ± 1.8 vs. 4.1 ± 3.3). The Pearson’s correlation coefficient was 0.969, indicating a strong correlation (Figure 4 and Table 3).

For the axillary lymph node analysis, two patients (patients one and four) with different nodes showed the greatest SUVmax for the ^18^F-FLT PET/CT and ^18^F-FDG PET/CT; consequently, the SUVmax of the corresponding lymph node was used for the analysis. The Shapiro–Wilk test showed that the SUVmax of the ^18^F-FDG PET/CT was normally distributed (*p* = 0.097) but not that of the ^18^F-FLT PET/CT (*p* = 0.000). The subsequent Wilcoxon signed-rank test showed no significant between-group difference (*p* = 0.246). The mean and standard deviation were 3.8 ± 6.3 for the ^18^F-FLT PET/CT and 4.8 ± 5.1 for the ^18^F-FDG PET/CT. The Pearson’s correlation coefficient was 0.999, indicating a strong correlation (Figure 4 and Table 3).

### 3.4. Patients with Prior Treatment before PET/CT

**Figure 5:**
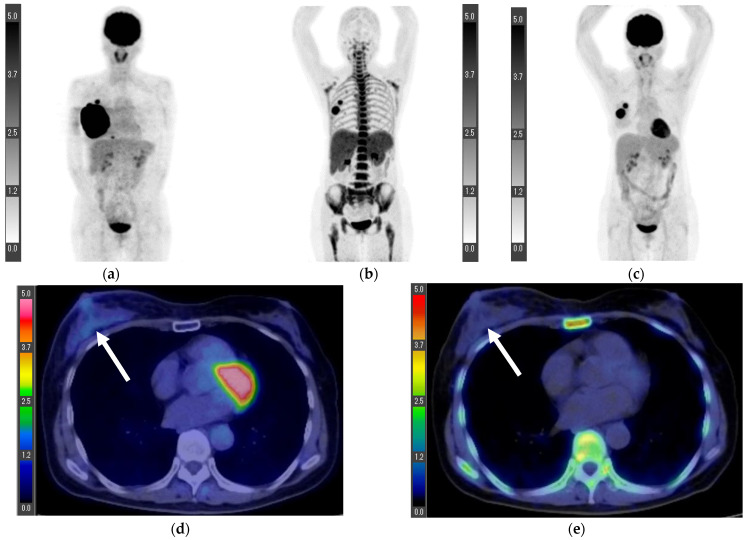
Maximum intensity projection (MIP) of ^18^F-FDG PET (**a**), MIP of ^18^F-FLT PET 7 months after chemotherapy (**b**), and MIP of ^18^F-FDG PET 4 days after the ^18^F-FLT PET (**c**). ^18^F-FDG accumulation increased in the right breast following chemotherapy ((**e**), arrow), whereas ^18^F-FLT accumulation was not evident ((**d**), arrow). Patient 7 (Figure 5).

Patient 7 was a 62-year-old woman diagnosed with right-sided breast cancer who only received chemotherapy. After five years, the initial ^18^F-FDG PET/CT showed a large right primary breast tumor, intramammary metastases, and multiple right axillary lymph node metastases (Figure 5). The SUVmax of the primary lesion was 22.0. Additional chemotherapy was provided. She then underwent ^18^F-FLT PET/CT seven months later, followed by a second ^18^F-FDG PET/CT four days thereafter. The SUVmax of the primary lesion reduced to 7.0 on the ^18^F-FLT PET/CT and 10.1 on the ^18^F-FDG PET/CT. On the second ^18^F-FDG PET/CT, the accumulation in the right breast was higher than that in the contralateral breast, with an SUVmax of 1.4. However, no increased accumulation was noted on the ^18^F-FLT PET/CT, with an SUVmax of 0.6. We concluded that the ^18^F-FDG accumulation in the right breast occurred secondary to inflammation. A subsequent biopsy revealed that her right-sided breast cancer was an invasive ductal carcinoma.

**Figure 6:**
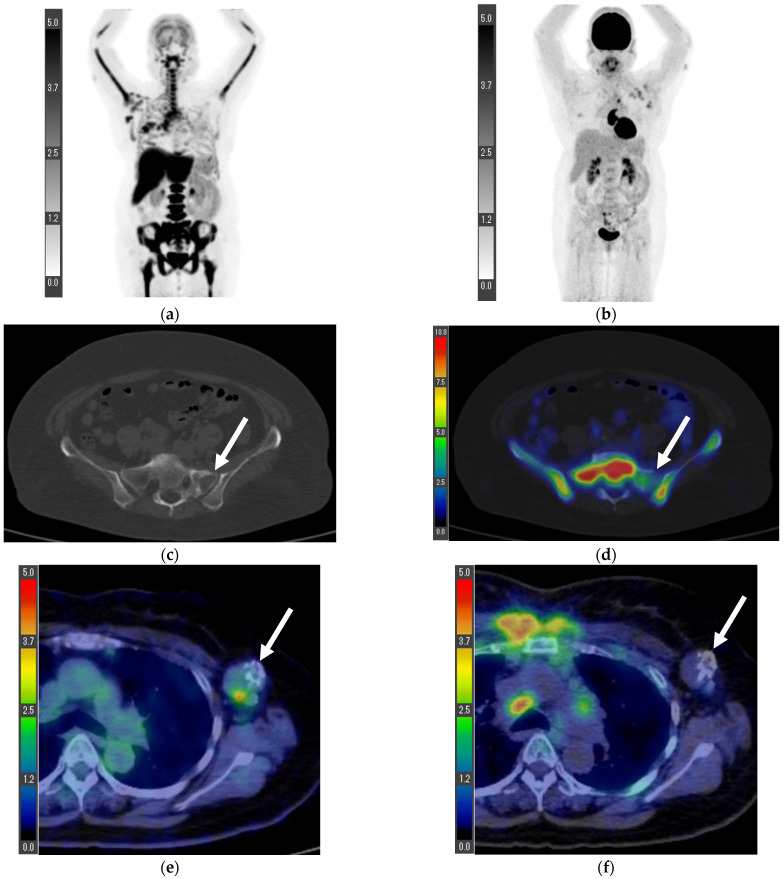
Maximum intensity projection (MIP) of ^18^F-FLT PET (**a**) and ^18^F-FDG PET 10 months prior (**b**). ^18^F-FLT accumulation within an osteolytic lesion ((**c**), arrow) on the left side of the sacrum was lower than that in the physiological bone marrow ((**d**), arrow). In addition, left axillary lymph node accumulation with coarse calcification was found on ^18^F-FDG PET/CT ((**e**), arrowhead); however, accumulation on ^18^F-FLT PET/CT did not increase ((**f**), arrowhead), suggesting inflammation. Patient 8 (Figure 6).

Patient 8 was a 51-year-old woman treated for left-sided breast cancer and multiple metastases for 7 years. She underwent multiple ^18^F-FDG PET/CTs. Her left breast resection revealed an invasive ductal carcinoma. Thereafter, ^18^F-FLT PET/CT was performed as the latest PET/CT (Figure 6); on the left side of the sacral bone, we found an osteolytic mass, which appeared metastatic. Considering that the lesion had lower ^18^F-FLT accumulation than the bone marrow, we concluded that the bone metastasis had less active cell proliferation than the bone marrow. This lesion was not diagnosed histologically, but it had progressed with other lesions on subsequent imaging follow-ups. The left axillary lymph nodes (which exhibited accumulation on the ^18^F-FDG PET/CT 10 months prior) displayed coarse calcification; therefore, it was difficult to identify whether the ^18^F-FDG accumulation reflected inflammation or viable cancer. Conversely, we observed no accumulation on the ^18^F-FLT PET/CT, despite no change in lymph node size. Therefore, cell proliferation was low in the left axillary lymph node with the ^18^F-FDG accumulation, thereby indicating inflammation.

## 4. Discussion

This study revealed that ^18^F-FLT accumulation secondary to primary breast cancer was lower than ^18^F-FDG accumulation in all cases, and significant differences were found in the SUVmax between these accumulations in treatment-naive patients. In patients with primary breast cancer and axillary lymph nodes, the SUVmax showed a strong correlation between the ^18^F-FLT PET/CT and ^18^F-FDG PET/CT. In particular, ^18^F-FLT PET/CT may be useful for estimating cell proliferation in lymph nodes and bone metastases, distinguishing such changes between inflammation and physiological accumulation.

^18^F-FLT is a radiolabeled imaging agent serving as the structural analog of the DNA constituent, thymidine [18,24]. The radiolabeling activity depends on DNA replication within the cells; hence, ^18^F-FLT uptake depends on the cells’ proliferative rate [18,24]. In contrast, ^18^F-FDG accumulation depends on glucose intake and reflects an increased metabolism and the Warburg effect [18]. Few studies have examined the differences between ^18^F-FLT and ^18^F-FDG accumulation in human breast cancer. For instance, in a study by Smyczek-Gargya et al., six patients with breast cancer received ^18^F-FLT PET/CT and ^18^F-FDG PET/CT within one week. Five of them possessed primary breast cancers with a lower SUVmax and SUVmean in the ^18^F-FLT PET/CT than in the ^18^F-FDG PET/CT, whereas the remaining patients demonstrated the opposite result; unfortunately, the reason for this discrepancy was not discussed [19]. In the present study, the SUVmax of ^18^F-FLT in the primary lesions averaged 2.1 (range, 0.6–4.8), whereas that of ^18^F-FDG averaged 4.1 (range, 1.5–8.4) among treatment-naive patients (patients 1–6). The SUVmax of ^18^F-FLT was approximately half that of ^18^F-FDG; this difference was statistically significant in patients 1–6. One esophageal cancer study showed that the ^18^F-FLT uptake was significantly lower than the ^18^F-FDG uptake, potentially as a result of the difference in biochemical and biological mechanisms between these two radiotracers during cell proliferation and differentiation [25]. Although low ^18^F-FLT accumulation might lead to an oversight, studies of head and neck squamous cell cancers have shown that the detection of primary lesions and metastatic lymph nodes was comparable between ^18^F-FLT PET/CT and ^18^F-FDG PET/CT [12,26]. Our present study also reported a strong correlation of the SUVmax between ^18^F-FLT and ^18^F-FDG in primary breast cancer and axillary lymph nodes.

Because ^18^F-FLT PET/CT reflects the cell proliferation cycle, several reports show an association with the Ki-67 labeling index, a pathological marker of cell proliferation [6,9,13]. The accumulation of ^18^F-FLT correlates with the Ki-67 labeling index [6,27]. In a meta-analysis that investigated the relationship between the SUVmax and the Ki-67 labeling index in breast cancer, ^18^F-FLT PET showed a higher correlation coefficient (*r* = 0.54) than ^18^F-FDG PET (*r* = 0.40) [28]. In our study, the higher the Ki-67 labeling index, the higher the ^18^F-FLT and ^18^F-FDG accumulation in surgical cases (patients 1–3). However, this relationship did not seem relevant in inoperable cases (patients 4–6). This discrepancy could be attributed to the fact that the Ki-67 labeling index was measured in a small number of biopsy specimens obtained from patients with inoperable diseases. In other words, estimating cell proliferation using ^18^F-FLT PET/CT might be helpful in measuring the accuracy of the Ki-67 labeling index in patients with inoperable diseases.

Our study yielded interesting results regarding lymph node accumulation. The lymph nodes with the greatest accumulation sometimes differed between the ^18^F-FLT PET/CT and the ^18^F-FDG PET/CT. In two patients, the SUVmax of ^18^F-FLT was higher than that of ^18^F-FDG. A study on primary colorectal cancer similarly concluded that the SUVmax of ^18^F-FLT was significantly lower than that of ^18^F-FDG in primary foci; however, the SUVmax of ^18^F-FLT was not always lower, and the SUVmax values of the two tracers were not significantly different in positive metastatic nodes [29]. The authors speculated that this finding was probably due to the different biochemical characteristics of the two tracers. Lymph nodes with higher ^18^F-FLT accumulation may have faster cancer cell proliferation. In thoracic esophageal squamous cell carcinoma, ^18^F-FLT PET/CT has a significantly higher specificity for diagnosing lymph node metastases than ^18^F-FDG PET/CT [16]; and in such cases, ^18^F-FLT PET/CT may be deemed more important than ^18^F-FDG PET/CT.

A few studies have evaluated distant metastatic lesions using ^18^F-FLT PET/CT. In patients four and eight, the bone metastases had a relatively low accumulation because of the high physiological ^18^F-FLT accumulation in the bone marrow. Therefore, on ^18^F-FLT PET/CT, bone metastases may demonstrate a lower accumulation than bone marrow; however, no similar reports exist. Interestingly, the lumbar metastasis in patient six showed ^18^F-FLT accumulation equivalent to that of bone marrow. In this case, concurrent CT might facilitate the detection of bone metastases.

Because physiological ^18^F-FLT accumulation in the brain is extremely low, brain metastases should be easily detected [30]; however, the present study had no patients with brain metastases. ^18^F-FLT PET can provide high specificity because ^18^F-FLT is less likely to accumulate in areas of inflammation secondary to cancer therapy [6,8,17]. Therefore, ^18^F-FDG accumulation and scarce ^18^F-FLT accumulation in the lymph nodes and breast are presumably the results of inflammation.

This study has several limitations. First, the sample size was small. Second, we did not control for surgical history, disease stage, or prior treatments. However, patients were divided into treatment-naive and non-treatment-naive groups before statistical analysis. Third, one patient did not undergo ^18^F-FDG PET/CT concurrently with ^18^F-FLT PET/CT. Fourth, the assessments performed by the nuclear medicine specialists were not consistent because the diagnoses were conducted prospectively. Finally, considering that breast cancer diagnoses were conducted before PET/CT, the influence of a prior biopsy was inevitable.

## 5. Conclusions

A strong correlation was found between the ^18^F-FLT PET/CT and ^18^F-FDG PET/CT uptake in primary breast tumors and axillary lymph nodes. Differences in the degree of breast cancer accumulation, lesions with the greatest accumulation, and physiological accumulation in organs reflected the different biochemical characteristics of ^18^F-FLT PET/CT and ^18^F-FDG PET/CT. Clinicians should be aware of these features when using ^18^F-FLT PET/CT to evaluate patients with breast cancer.

## Figures and Tables

**Table 1:** Ages of treatment-naive patients before positron emission tomography/computed tomography and time series of each examination.

	Age (Years)	^18^F-FLT PET/CT (Days) *	^18^F-FDG PET/CT (Days) *	Surgery (Days) *
Patient 1	49	35	33	56
Patient 2	72	27	20	41
Patient 3	73	21	18	27
Patient 4	68	22	19	-
Patient 5	71	7	19	-
Patient 6	72	15	40	-
Patient 7	63	-	-	-
Patient 8	52	-	-	-

^18^F-FLT PET/CT, ^18^F-fluorothymidine positron emission tomography/computed tomography; ^18^F-FDG PET/CT, ^18^F-fluorodeoxyglucose positron emission tomography/computed tomography. * Days since biopsy.

**Table 2:** Positron emission tomography/computed tomography (PET/CT) and pathology results of treatment-naive patients before PET/CT.

Patient	Primary FLT	Primary FDG	LN FLT	LN FDG	Stage	Histological Diagnosis	Nuclear Grade	ER	PR	HER2	Ki-67 (%)
1	1.0	1.8	1.6 *	3.4 *	T1cN2aM0 **	Invasive ductal carcinoma	1	+	+	−	1.0
2	0.6	1.6	0.7	0.8	T1bN0M0 **	Invasive ductal carcinoma	1	+	+	−	6.7
3	2.2	3.1	0.5	0.6	T1cN0M0 **	Invasive ductal carcinoma	2	+	+	−	24.6
4	0.4	1.5	16.7 *	14.2 *	T1bN3aM1 *** (Lymph node and bone metastases)	Invasive ductal carcinoma	Data loss	+	+	−	Data loss
5	3.8	8.3	1.8	6.3	T2N3bM0 ***	Invasive ductal carcinoma	1	+	+	−	25
6	4.8	8.4	1.4	3.2	T4bN1M1 *** (Lumbar vertebra metastasis)	Invasive ductal carcinoma	1	+	+	−	8.9
7 ****	7.0	10.1	1.2	0.7	T4bN0M0	Invasive ductal carcinoma	Data loss	−	−	+	59
8 *****	-	-	1.1	4.1	N3bM1 (Lymph node, lung, and bone metastases)	Invasive ductal carcinoma	3	+	−	+	29.3

Primary FLT, maximum standardized uptake value (SUVmax) of primary breast cancer on ^18^F-fluorothymidine positron emission tomography/computed tomography (^18^F-FLT PET/CT); primary FDG, SUVmax of primary breast cancer on ^18^F-fluorodeoxyglucose positron emission tomography/computed tomography (^18^F-FDG PET/CT); LN FLT, SUVmax of the axillary lymph node on ^18^F-FLT PET/CT; LN FDG, SUVmax of the axillary lymph node on ^18^F-FDG PET/CT; ER, estrogen receptor; PR, progesterone receptor; HER2, human epidermal growth factor 2; +, positive; −, negative. * The axillary lymph node that showed the greatest accumulation differed between ^18^F-FLT PET/CT and ^18^F-FDG PET/CT. The SUVmax on the ^18^F-FLT PET/CT showed lymph node values corresponding to those on the ^18^F-FDG PET/CT. The SUVmax of the lymph node with the greatest accumulation on the ^18^F-FLT PET/CT was 3.1 for patient 1 and 30.0 for patient 4. ** Pathological stage determined via surgery. *** Clinical stage determined via biopsy and PET/CT. **** SUVmax, stage, histological diagnosis, and immunostaining results are all post-chemotherapy data. ***** SUVmax of the axillary lymph nodes showed recent ^18^F-FLT PET/CT and ^18^F-FDG PET/CT 10 months prior. Histological diagnosis and immunostaining results were obtained using surgical specimens after chemotherapy.

**Table 3:** Results of the statistical analysis of the SUVmax on the ^18^F-FLT PET/CT and ^18^F-FDG PET/CT for treatment-naive patients.

	Significant Difference	Correlation Coefficient
Primary breast cancer	*p* = 0.031 *	0.969
Axillary lymph node	*p* = 0.246 **	0.999

SUVmax, maximum standardized uptake value; ^18^F-FLT PET/CT, ^18^F-fluorothymidine positron emission tomography/computed tomography; ^18^F-FDG PET/CT, ^18^F-fluorodeoxyglucose positron emission tomography/computed tomography. * Result of the two-tailed paired *t*-test. ** Result of the Wilcoxon signed-rank test.

## Data Availability

No new data were created or analyzed in this study. Data sharing is not applicable to this article.
